# HE2Gene: image-to-RNA translation via multi-task learning for spatial transcriptomics data

**DOI:** 10.1093/bioinformatics/btae343

**Published:** 2024-06-05

**Authors:** Xingjian Chen, Jiecong Lin, Yuchen Wang, Weitong Zhang, Weidun Xie, Zetian Zheng, Ka-Chun Wong

**Affiliations:** Cutaneous Biology Research Center, Massachusetts General Hospital, Harvard Medical School, Boston, MA 02129, USA; Department of Computer Science, City University of Hong Kong, Kowloog Tong 999077, Hong Kong SAR; Molecular Pathology Unit, Center for Cancer Research, Massachusetts General Hospital, Department of Pathology, Harvard Medical School, Boston, MA 02129, USA; Department of Computer Science, The University of Hong Kong, Pokfulam 999077, Hong Kong SAR; Department of Computer Science, City University of Hong Kong, Kowloog Tong 999077, Hong Kong SAR; Department of Computer Science, City University of Hong Kong, Kowloog Tong 999077, Hong Kong SAR; Department of Computer Science, City University of Hong Kong, Kowloog Tong 999077, Hong Kong SAR; Department of Computer Science, City University of Hong Kong, Kowloog Tong 999077, Hong Kong SAR; Department of Computer Science, City University of Hong Kong, Kowloog Tong 999077, Hong Kong SAR; Shenzhen Research Institute, City University of Hong Kong, Shenzhen 518057, China

## Abstract

**Motivation:**

Tissue context and molecular profiling are commonly used measures in understanding normal development and disease pathology. In recent years, the development of spatial molecular profiling technologies (e.g. spatial resolved transcriptomics) has enabled the exploration of quantitative links between tissue morphology and gene expression. However, these technologies remain expensive and time-consuming, with subsequent analyses necessitating high-throughput pathological annotations. On the other hand, existing computational tools are limited to predicting only a few dozen to several hundred genes, and the majority of the methods are designed for bulk RNA-seq.

**Results:**

In this context, we propose HE2Gene, the first multi-task learning-based method capable of predicting tens of thousands of spot-level gene expressions along with pathological annotations from H&E-stained images. Experimental results demonstrate that HE2Gene is comparable to state-of-the-art methods and generalizes well on an external dataset without the need for re-training. Moreover, HE2Gene preserves the annotated spatial domains and has the potential to identify biomarkers. This capability facilitates cancer diagnosis and broadens its applicability to investigate gene-disease associations.

**Availability and implementation:**

The source code and data information has been deposited at https://github.com/Microbiods/HE2Gene.

## 1 Introduction 

The development of sequencing technologies from bulk RNA-seq to single-cell RNA-seq (scRNA-seq) has significantly advanced the understanding of gene expression at the cellular level (Chen et al. 2019). However, existing scRNA-seq technologies cannot preserve the spatial location of cells due to their destructive nature; therefore, it is difficult to understand cellular interactions in their native tissue context (Williams et al. 2022). In recent years, a multitude of spatially resolved transcriptomics technologies have been developed (Strell et al. 2019). These technologies facilitate the concurrent visualization of spatial locations and gene expression patterns within complex tissues. They can be broadly categorized into imaging-based methods and sequencing-based methods (Strack 2022). Imaging-based methods such as single-molecule RNA fluorescence *in situ* hybridization (smFISH), multiplexed error-robust FISH (MERFISH), and sequential FISH (seqFISH) provide higher sequencing resolutions (Tian et al. 2023); however, they can only measure a limited number of genes, which may not be sufficient for studying complex diseases that involve various gene expression patterns (Lein et al. 2017). Sequencing-based methods such as spatial transcriptomics (ST), Visium, and Slide-seq have higher throughput, although they are not measured at single-cell resolution (Ståhl et al. 2016). However, both of them remain expensive and time-consuming, with subsequent analyses necessitating high-throughput pathological annotations from experts.

Fortunately, sequencing-based methods are usually compatible with H&E-stained whole-slide images (Strell et al. 2019). For example, Visium scans the H&E-stained image of a tissue section to simultaneously measure tissue morphology and spatial transcriptome (Ståhl et al. 2016). Consequently, some computational methods have been developed to quantify gene expressions from tissue images. This advancement significantly enhances our understanding of normal development and disease pathology, improves analysis efficiency, and reduces sequencing costs (Kleino et al. 2022). For example, Schmauch et al. (2020) introduced HE2RNA to systematically predict bulk RNA-seq profiles from whole-slide images. He et al. (2020) proposed ST-Net to predict spot gene expression from H&E-stained images of breast cancer patients. Pang et al. (2021) developed HisToGene, which predicts super-resolution gene expression by integrating spot position information and Vision Transformer. Zeng et al. (2022) presented Hist2ST, which combined ConvMixer, Transformer, and graph neural networks for gene expression inference and spatial region identification. While these computational methods have demonstrated promising breakthroughs, they also have some limitations. For example, while HE2RNA was designed for bulk RNA-seq, its generalization to single-cell and spatial RNA-seq remains speculative (Schmauch et al. 2020). HisToGene and Hist2ST can only accept whole-slide images as input, which poses difficulties when implementing them on single-spot patches (Pang et al. 2021, Zeng et al. 2022). In addition, both methods were developed by incorporating the spot position information at a fixed scale, limiting their generalization to new scenarios with different imaging resolutions. Furthermore, ST-Net focuses on investigating and modeling only the top few hundred genes of interest (He et al. 2020). This results in the loss of information for tens of thousands of missing genes, indicating that there is still room for improvement.

Recently, several computational methods have been developed to utilize hundreds of landmark genes as input to predict the remaining thousands of unmeasured genes (Jeon et al. 2022). For example, Chen et al. proposed a multi-task multi-layer feedforward neural network D-GEX to infer the expression of 9520 target genes from 943 landmark genes (Chen et al. 2016). Wang et al. developed a conditional generative adversarial network to estimate the target gene expressions using the same datasets (Wang et al. 2018). These studies demonstrated that gene co-expression patterns and gene–gene interactions can be utilized to predict the expression of tens of thousands of genes from just hundreds of genes (Subramanian et al. 2017). On the other hand, there have been studies for utilizing image or gene expression data as features to build machine-learning models for survival prediction, tumor identification, and disease stratification (Pineda et al. 2016, Duran-Lopez et al. 2020, Wulczyn et al. 2020). These studies have demonstrated the correlation between gene expression and tissue morphology with the human phenotype. The reciprocal influence between gene expression and tissue morphology provides potential biomarkers for various cancer types.

Motivated by the aforementioned studies, we propose HE2Gene, a deep learning-based method that models gene–gene correlations and includes pathological annotations to predict gene expressions from H&E-stained images. By leveraging a multi-task learning framework, we integrated information on non-target genes, pathological annotations, and spatial dependencies among neighboring patches to facilitate and improve the prediction of hundreds of target genes. To the best of our knowledge, this is the first deep learning-based method that can simultaneously predict spot-level gene expressions and pathological annotations. The proposed non-target gene inference task facilitates the prediction of tens of thousands of remaining genes, while the novel spatial constraint incorporates patch-based spatial dependencies between pathological annotations and tissue morphology. By including pathological annotations, HE2Gene can be easily extended for tumor detection tasks. We present the materials, methods, and experimental results of HE2Gene in the subsequent sections.

## 2 Materials and methods

### 2.1 Datasets

**Figure 1.**
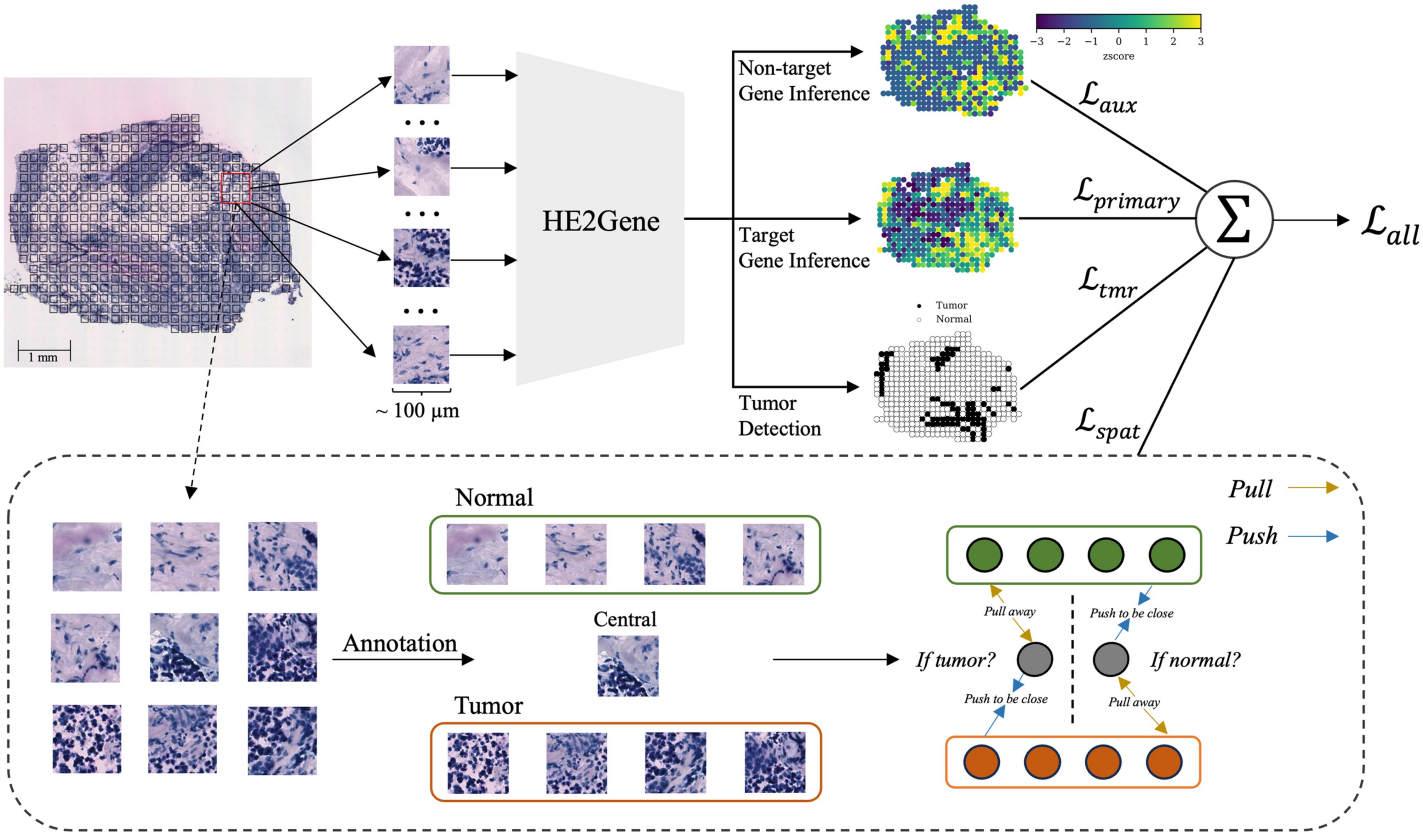
Overview of HE2Gene. (Upper) The whole-slide image is segmented into hundreds of spots using the coordinates obtained from spatial transcriptomics protocols. Spot images are then fed into HE2Gene for gene expression prediction and pathological annotation using multi-task learning. HE2Gene has three prediction tasks, which are to predict the target gene expressions, non-target gene expressions, and pathological annotations, respectively. (Bottom) HE2Gene also includes a spatial-aware loss function to incorporate patch-based spatial dependencies between pathological annotations and tissue morphology. Given a central patch and its neighboring patches, the spatial loss minimizes the discrepancy in the predicted gene expressions between patches with the same pathological annotations.

We utilized two recently published breast cancer datasets measured by ST technologies (Andersson et al. 2020, He et al. 2020). The first dataset (HBCIS) consists of 23 breast cancer patients with 5 different subtypes (Luminal A, Luminal B, Triple-Negative, HER2-positive Luminal, and HER2-positive Non-luminal) (He et al. 2020). Each patient has two to three replicates with H&E-stained tissue images (scanned at ×20 magnification) and the corresponding spatial gene expression data. There are hundreds of spots (ranging from 256 to 712) captured on each tissue slide. Each spot has a diameter of 100 μm, and the spots are arranged in a grid with a center-to-center distance of 200 μm (see Fig. 1). The distance between adjacent spots is about 100 μm. The number of cells in each spot varied from one to tens in different regions of the tissues. The spatial coordinate in the slide and the pathological annotations made by the pathologists (tumor or normal) are provided for each spot. In total, 30 612 spots in 68 tissue slides from 23 breast cancer patients were observed across the whole dataset, and each spot was measured by 26 949 gene expressions. Detailed sample processing steps can be found in previous studies (He et al. 2020), where breast cancer biopsies were collected and handled as described, and the RNA-seq of the barcoded libraries and subsequent analysis from tissue sections were performed using standardized protocols (Salmén et al. 2018).

The second dataset (HER2+) serves as an independent external test and for comparison purposes (Andersson et al. 2020). It originates from a study on human HER2-positive breast tumors and was measured using the 10× Visium platform. This dataset comprises 36 tissue sections from 8 patients and the sections were scanned at ×20 magnification. We preprocessed the HER2+ dataset following the previous studies (Pang et al. 2021, Zeng et al. 2022) and only selected the slides with available pathological annotations (including invasive cancer, breast glands, immune infiltrate, cancer *in situ*, connective tissue, adipose tissue, and undetermined), resulting in a total of 3481 spots and 17 844 genes from eight patients. For both datasets, each patient’s data includes gene expression data, spatial coordinates, and whole-slide images. The gene expression data are stored in an *N *×* M* matrix where *N* is the number of spots and *M* is the number of genes. The spatial coordinates of the spots are stored in a *N *×* *2 matrix indicating the (*x*, *y*) location of each spot on the slide. There are hundreds of spots captured on each slide, and each spot has a size of around 100 × 100 ${\mu m}^{2}$, corresponding to a patch of approximately 150 × 150 pixels. Therefore, we segmented a square patch for each spot by its spatial coordinate with a window size of 150 and resized it to a 224 × 224-pixel RGB image to use as input for the deep neural network, since a 224 × 224-pixel image is the standard and most popular input size for convolutional neural networks (He et al. 2016).

Due to the heterogeneity of image and gene expression data, as well as the inherent batch effects present in ST experiments, several quality control procedures were implemented for each sample across all patients. For image data, we excluded spots where the image was too close to or beyond the boundaries of the whole slide. For sequencing data, spots with fewer than 200 unique molecular identifiers (UMIs), more than 20 000 UMIs, over 5% mitochondrial genes, and fewer than 200 detected genes were excluded. Genes detected in fewer than three spots were also excluded. Spot images were initially normalized by dividing each pixel value by 255, followed by subtracting the mean and then dividing by the standard deviation of the pixel values in each channel. The gene expression data is normalized by first dividing by the total counts in each spot and multiplying by a scale factor of 10 000, then the log value of the normalized counts is taken to bring the values into a reasonable range. A pseudo count is added before transformation to prevent the use of zeros in the logarithm. These quality control procedures and normalization techniques improved the accuracy of downstream analyses and ensured the reliability of the results.

### 2.2 Overview of HE2Gene

HE2Gene is designed to infer gene expressions and pathological annotations of tissue samples from their corresponding H&E-stained images. To accomplish this, we utilized a collection of training samples. Each sample is associated with a whole-slide H&E-stained image, the spatial coordinates of each spot on the image, and the corresponding spot-level gene expression and pathological annotations. In the inference stage, by integrating the predicted gene expressions and pathological annotations with the input H&E-stained images, we can establish a comprehensive tissue data modality. This approach merges spatial and genomic-scale data with information on tumor phenotype and morphology, providing a holistic view of the tissue samples. The overview of HE2Gene is depicted in Fig. 1.

### 2.3 Multi-task learning

Recently, multi-task learning has become popular in various fields, such as computer vision and natural language processing (Zhang and Yang 2018). Unlike training separate models for each task, multi-task learning enables a model to learn a shared representation for multiple tasks simultaneously. This approach improves generalization by exploiting the similarities and differences among the related tasks. Moreover, knowledge transfer between tasks can facilitate faster convergence during training. In this study, we developed HE2Gene based on the multi-task learning framework. HE2Gene integrates a primary task for target gene inference, two auxiliary tasks for non-target gene inference and tumor detection, and a spatial-aware constraint for modeling patch-based spatial dependencies (Fig. 1). The detailed descriptions of each task and the associated loss function are introduced in the subsequent sections.

#### 2.3.1 Target and non-target gene inference tasks

In this study, gene expression inference is treated as a multi-output regression problem, where the goal is to predict the expressions of many genes simultaneously. Existing methods for H&E-based gene expression prediction in ST, such as ST-Net (He et al. 2020), HisToGene (Pang et al. 2021), and Hist2ST (Zeng et al. 2022), have primarily focused on the top several hundred genes of interest for model construction and prediction, resulting in the loss of information from tens of thousands of remaining genes. Conversely, there are studies that have demonstrated that a set of approximately 1000 landmark genes have been identified that contain about 80% information of the whole genome and can be used to predict the expression of remaining genes (Chen et al. 2016, Wang et al. 2018). These findings motivated us to consider the unused genes as non-target genes and to construct an auxiliary task. By utilizing tissue context and non-target gene expression data, we aim to improve the performance of our primary task, which is the prediction of the 250 target genes with the highest mean expression. Herein, we define two tasks corresponding to the minimization of a primary loss $L_{\text{primary}}$ for target gene inference and an auxiliary loss $L_{\text{auxiliary}}$ for non-target gene inference, the underlying intuition is that minimizing the auxiliary loss will lead to more meaningful representations in the shared layers and these representations can be leveraged by the layers specific to the primary task. The loss function for the primary target gene inference task can be defined as Equation (1):

(1) $$L_{\text{prim}}=\frac{1}{mN}\sum_{i=1}^{N}\sum_{j=1}^{m}{\left(g_{i(j)}-{\hat{g}}_{i(j)}\right)}^{2},$$

where *m* is the number of target genes and *N* is the number of spots. *g* and $\hat{g}$ represent the ground truth and predicted gene expression, respectively. Herein, the mean squared error (MSE) is used to measure the difference. Similarly, the loss function for the auxiliary non-target gene inference task can be formulated as Equation (2):

(2) $$L_{\text{aux}}=\frac{1}{N(M-m)}\sum_{i=1}^{N}\sum_{j=m+1}^{M}{\left(g_{i(j)}-{\hat{g}}_{i(j)}\right)}^{2},$$

where *M* represents the number of all genes, *M−m* defines the number of selected non-target genes. We employed the hard parameter-sharing strategy of the multi-task learning framework.

#### 2.3.2 Tumor detection task

The differences in gene expression patterns between cancer and normal tissues are crucial for understanding cancer pathogenesis. Existing studies have demonstrated that cancer patients can be classified based on the expression levels of certain genes from their pathological tissues (Hou et al. 2010). By leveraging existing pathological annotations, we proposed to incorporate tumor detection as another auxiliary task to improve the performance of target gene inference. This approach can lead to faster convergence of gene inference tasks and a deeper understanding of the underlying relationship between gene expression and cancer. The loss function for the tumor detection task can be defined as Equation (3):

(3) $$L_{\text{tmr}}=-\frac{1}{N}\sum_{i=1}^{N}\left[y_{i}\;\text{log}\;{\hat{y}}_{i}+(1-y_{i})\;\text{log}(1-{\hat{y}}_{i})\right],$$

where *N* is the number of spots, *y* and $\hat{y}$ represent the ground truth and predicted pathological annotations, respectively. Herein, the task is to predict whether a spot image is cancer or normal tissue, and cross-entropy is utilized as the loss function. The predicted pathological annotations can be utilized to compare with the predicted gene expressions, exploring potential cancer-related biomarkers for subsequent ST analyses. Tasks such as cell-type annotation or cancer metastasis detection could also be employed.

#### 2.3.3 Spatial-aware constraint

Recent studies on image-based cancer detection tasks have revealed that when a patch in a slide is labeled as a tumor region, its neighboring patches also have a high probability of being labeled as tumors (Wang et al. 2019). In this premise, we assume that adjacent patches with the same annotation in whole-slide images tend to have similar gene expressions. This presumption is based on the understanding that overexpression or underexpression of certain genes can precipitate disease states (Kastora et al. 2022). Moreover, it has been demonstrated that some genes serve as biomarkers for specific diseases (Tripathi et al. 2011, Zhang et al. 2022), further supporting the notion that spatial proximity in tissue samples correlates with gene expression patterns related to disease conditions. Therefore, we incorporated the spatial relationships between the central patch and its eight neighboring patches (arranged in a 3 × 3 grid) into a loss function. Specifically, given a random central spot and its neighboring spots, if they share the same pathological annotations, their gene expression predictions should be as similar as possible. Herein, assume the classifier is *η*, a novel spatial loss function is defined as Equation (4):

(4) $$L_{\text{spat}}=\frac{1}{K}\sum_{i=1}^{K}\sum_{j=1}^{N}w_{i,j}{\left(\eta \left(x_{i}\right)-\eta \left(x_{j}\right)\right)}^{2},$$

where *x_i_* is the central spot and $x_{j}(j=1,2,\ldots ,N,N\leq 8)$ is its eight neighboring spots, *K* is the number of neighboring spots with the same annotation as the central spot, $w_{i,j}$ is a weight term defined as Equation (5):

(5) $$w_{i,j}=\{\begin{array}{cc} 1, & \text{if}\;y_{i}=y_{j}\\ 0, & \text{otherwise} \end{array},$$

where *y_i_* and *y_j_* are the corresponding annotations of the central spot and its neighboring spots. By introducing spatial loss, neighboring spots with the same annotation are forced to have similar predicted gene expressions. Finally, the entire loss function can be formulated as Equation (6):

(6) $$L_{\text{all}}=L_{\text{prim}}+\left(\lambda L_{\text{aux}}+\beta L_{\text{tmr}}+\gamma L_{\text{spat}}\right),$$

where $L_{\text{prim}},\;L_{\text{aux}},\;L_{\text{tmr}}$, and $L_{\text{spat}}$ represent the losses for the primary target gene inference task, auxiliary non-target gene inference task, tumor detection task, and spatial-aware constraint, respectively. We added additional weights *λ*, *β*, and *γ* for each loss to achieve the optimal performance.

## 3 Results

### 3.1 HE2Gene with multi-task learning significantly facilitates spatial gene inference

Due to the limited training data, we adapted an ImageNet pre-trained ResNet-50 (He et al. 2016) as the image encoder and fine-tuned all the convolutional layers for the gene expression inference and tumor detection tasks. ResNet-50 architecture is popular as the backbone in various computer vision tasks such as image classification, object detection, and semantic segmentation (Liu et al. 2022). There is a separate fully connected layer for each prediction task at the end of the convolutional backbone. Following the previous study (He et al. 2020), the primary task is to predict the top 250 genes with the highest mean gene expressions. This is because certain genes demonstrate very low expression levels (as shown in Supplementary Fig. S1), leading to a low signal-to-noise ratio. Such conditions present considerable challenges for achieving accurate predictions. The auxiliary tasks are to predict the expressions of the remaining 19 699 non-target genes and tumor detection. The detailed experimental settings are provided in Supplementary material. We listed the prediction results of the gene inference task and pathological annotation task in Table 1.

**Table 1.** Evaluation of spatial gene expression inference and pathological annotation for the HBCIS dataset.

Method	Target gene inference (250)		Non-target gene inference (19699)		Pathological annotation	
	aMSE ↓	aPCC ↑	aMSE ↓	aPCC ↑	ACC ↑	AUC ↑
Random	5.4294	0.0013	0.3272	−0.0001	0.4974	0.5005
Baseline (Prim)	1.3732	0.1349				
W/Aux	1.3318	0.1602	0.1727	0.0455		
W/Tmr	**1.3226**	0.1482			0.8218	0.9261
W/Spat	1.3396	0.1503				
HE2Gene-Base	1.3336	0.1774	0.1730	0.0417	**0.8459**	0.9315
HE2Gene-Spat	1.3273	**0.1818**	**0.1723**	**0.0521**	0.8329	**0.9374**

*Note*: aMSE represents the average mean square error, aPCC represents the average Pearson correlation coefficient, ACC represents the accuracy, and AUC represents the area under the receiver operating characteristic (ROC) curve. Bold values represent the best performance. “Random” refers to the baseline model without training. “Baseline (Prim)” represents the baseline model trained with the primary target gene inference task. “W/Aux” refers to the baseline model incorporating an auxiliary non-target gene inference task. “W/Tmr” denotes the baseline model integrating a tumor detection task. “W/Spat” represents the baseline model including the spatial-aware constraint. “HE2Gene-Base” refers to the baseline model trained with both non-target gene inference and tumor detection tasks, and “HE2Gene-Spat” denotes the baseline model trained with both auxiliary tasks and the spatial-aware constraint. See Supplementary Fig. S2 for detailed bar charts with error bars.

We observed that the proposed multi-task learning framework (HE2Gene-Base and HE2Gene-Spat) achieved substantial improvements compared to the baseline model with the primary task [Baseline (Prim)] and models trained separately with auxiliary tasks and spatial loss (W/Aux, W/Tmr, and W/Spat). Specifically, models incorporating a single auxiliary task and spatial loss (W/Aux, W/Tmr, and W/Spat) showed a 2%–4% increase in aPCCs compared to the baseline model [Baseline (Prim)]. Moreover, HE2Gene-Base and HE2Gene-Spat models demonstrated a 2%–3% improvement in aPCCs over models trained separately with auxiliary tasks and spatial loss (W/Aux, W/Tmr, and W/Spat), and a 5%–6% improvement compared to the baseline model [Baseline (Prim)]. We attribute the improvement to the gene–gene interactions between target genes and non-target genes, exploiting the similarities and differences in different tasks to learn a general representation. In addition, the spatial loss (W/Spat) achieves higher correlations by considering the spatial relationships between adjacent patches. It is notable that the improvement from the model with the pathological annotation task (W/Tmr) was not significant. We infer that it may be due to the binary pathological annotations lacking sufficient information compared to high-dimensional gene expression data.

In addition, we showed the distribution of the spatial correlation coefficients of genes across different baselines in Supplementary Fig. S3. It can be observed that for both target gene inference and non-target gene inference tasks, our HE2Gene can consistently predict most of the genes with positive correlations, as compared to the baseline model [Baseline (Prim)]. We also listed the prediction results of the top five genes that exhibit the largest expression differences between tumor and normal tissue spots (see Supplementary Table S1). These genes were selected by calculating the average gene expression of 250 target genes in both tumor and normal tissue spots across all patients and identifying the top five with the greatest differences in average expression. The selected genes—FASN, ACTG1, PTMA, GNAS, and HSP90AB1—have all been previously identified as known cancer biomarkers (Tripathi et al. 2011, Lin et al. 2020, Jiang et al. 2021, Kastora et al. 2022, Zhang et al. 2022). The positive predictive performance of HE2Gene for these genes indicates a higher correlation between biomarker genes and tissue morphology. The improvements in both ACC and AUC for tumor detection tasks, as displayed in Table 1, also underscore the potential of HE2Gene for cancer diagnosis.

### 3.2 HE2Gene accurately detects tumors and identifies potential biomarkers

**Figure 2.**
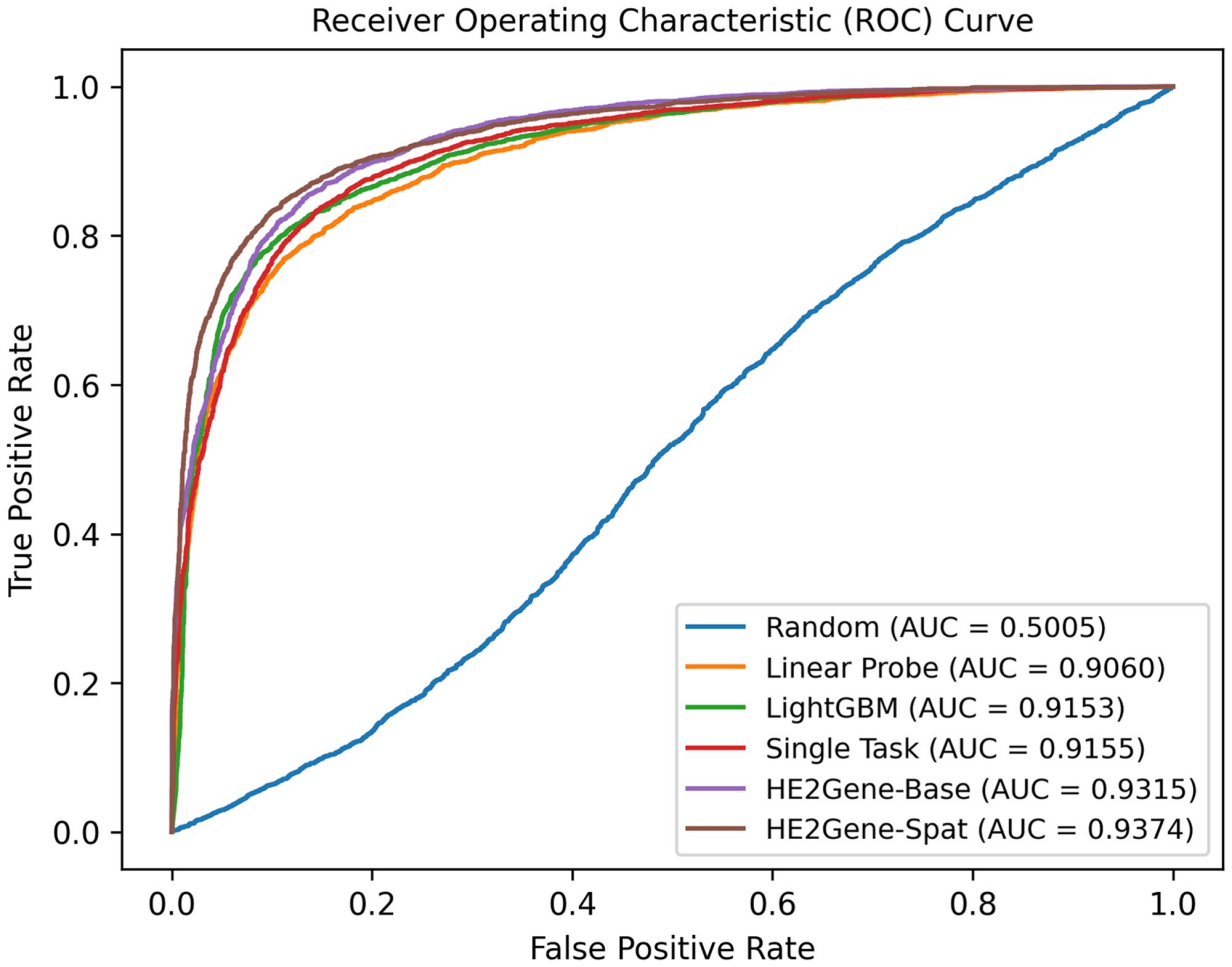
The mean AUCs on the tumor detection task are reported. “Random” means to predict without training. “Linear Probe” and “LightGBM” refer to extracting the image features from the pre-trained ResNet-50 model as the input for a linear classifier and a LightGBM for training. “Single Task” means to separately train the model for the pathological annotation task.

To further explore HE2Gene’s potential for cancer diagnosis, we compared its tumor detection performance with several benchmarks. As we can observe from Fig. 2 and Supplementary Fig. S4, both the AUC and the area under the precision-recall curve (AUPRC) of HE2Gene display 1%–4% improvements with respect to the single-task linear classifier, tree-based LightGBM classifier, and deep learning models, demonstrating the efficiency of HE2Gene and its potential for cancer diagnostic tasks. Specifically, compared with the single-task baseline model (Single Task), HE2Gene achieved 2%–3% improvements on both AUC and AUPRC, which supports the previous conclusion that gene expression information is more informative and can be utilized to facilitate tumor detection tasks.

To further investigate whether the predictions of HE2Gene can be utilized to recover pathological domains and identify potential biomarkers, we visualized and compared the original image and gene expression features with those extracted by HE2Gene for a test patient with HER2-positive breast cancer (as shown in Supplementary Fig. S5). We can observe that both variants of HE2Gene have learned more meaningful and discriminatory representations for tumor detection. Notably, while the original gene expression features could not distinguish between normal and cancer tissues, the refined features from HE2Gene exhibited a clear decision boundary. We infer the reason could be that the multi-task framework of HE2Gene captured the correlation between pathological annotation and gene expression. This integration helps to correct the noise and batch effect in the original gene expression (as evidenced in Supplementary Fig. S6), thereby enhancing its efficacy for the tumor detection task.

We also evaluated the performance of tumor detection classifiers trained on different data modalities, including images, gene expressions, and their combinations, to determine which modality yields the best results. Specifically, we divided the spots in test patients using an 8:2 ratio into training and test sets, respectively, and used a random forest as the classifier. Surprisingly, in most cases, the features extracted by HE2Gene, both singly and combined, provided better performance (see Supplementary Fig. S7). Furthermore, we plotted Venn diagrams to illustrate the overlaps of the top 30 and top 100 informative features extracted by the random forests trained on original and HE2Gene-predicted gene expression features. Our comparison of the informative gene lists revealed that all six genes identified by both HE2Gene-Base (RPL34, RPL36A, DDX5, RPL5, IFI6, and RPL4) and HE2Gene-Spat (TPT1, MYL9, RPS24, PTMA, FLNA, and RPL28) are corroborated by existing cancer studies (Yang et al. 2016; Alshabi et al. 2019; Ma et al. 2022; Lv and Li 2023; Lv et al. 2023). However, for the Ground Truth (original gene expression features), we could not find supporting studies for 10 of the 18 identified genes. This underscores HE2Gene’s potential in identifying biomarkers for tumor detection. Notably, there are only three overlapping genes in the non-target gene list, which could be attributed to their sparse expressions and the resulting low signal-to-noise ratio. Furthermore, since our primary focus is on target genes, the non-target genes may not hold significant interest.

### 3.3 HE2Gene consistently outperforms the state-of-the-art methods for gene expression prediction

To quantify the prediction performance of HE2Gene, we compared it with several deep learning-based benchmarks: HE2RNA (Schmauch et al. 2020), ST-Net (He et al. 2020), HisToGene (Pang et al. 2021), and Hist2ST (Zeng et al. 2022). HE2RNA is recognized as the state-of-the-art method for predicting bulk RNA-seq from whole-slide images, while ST-Net is the first benchmark for spatial gene inference from H&E-stained images. HisToGene and Hist2ST both integrated Vision Transformer and spot position information to predict gene expressions. Herein, we generalized HE2RNA to be applicable for spatial gene inference. Specifically, we adopted the same pre-trained model, classification head, and training strategy to retrain HE2RNA on our dataset. For ST-Net, HisToGene, and Hist2ST, we implemented them using the suggested optimal hyperparameters in the original studies. In addition, we implemented three other machine learning-based benchmarks: k-nearest neighbors (k-NN), logistic regression (Linear Probe), and random forest. The results were obtained by using an ImageNet pre-trained ResNet-50 to extract image features for gene expression inference. To ensure a fair comparison, all benchmark methods have been rigorously evaluated using the identical evaluation scheme applied to HE2Gene. The implementation details can be found in experimental settings in Supplementary material.

**Figure 3.**
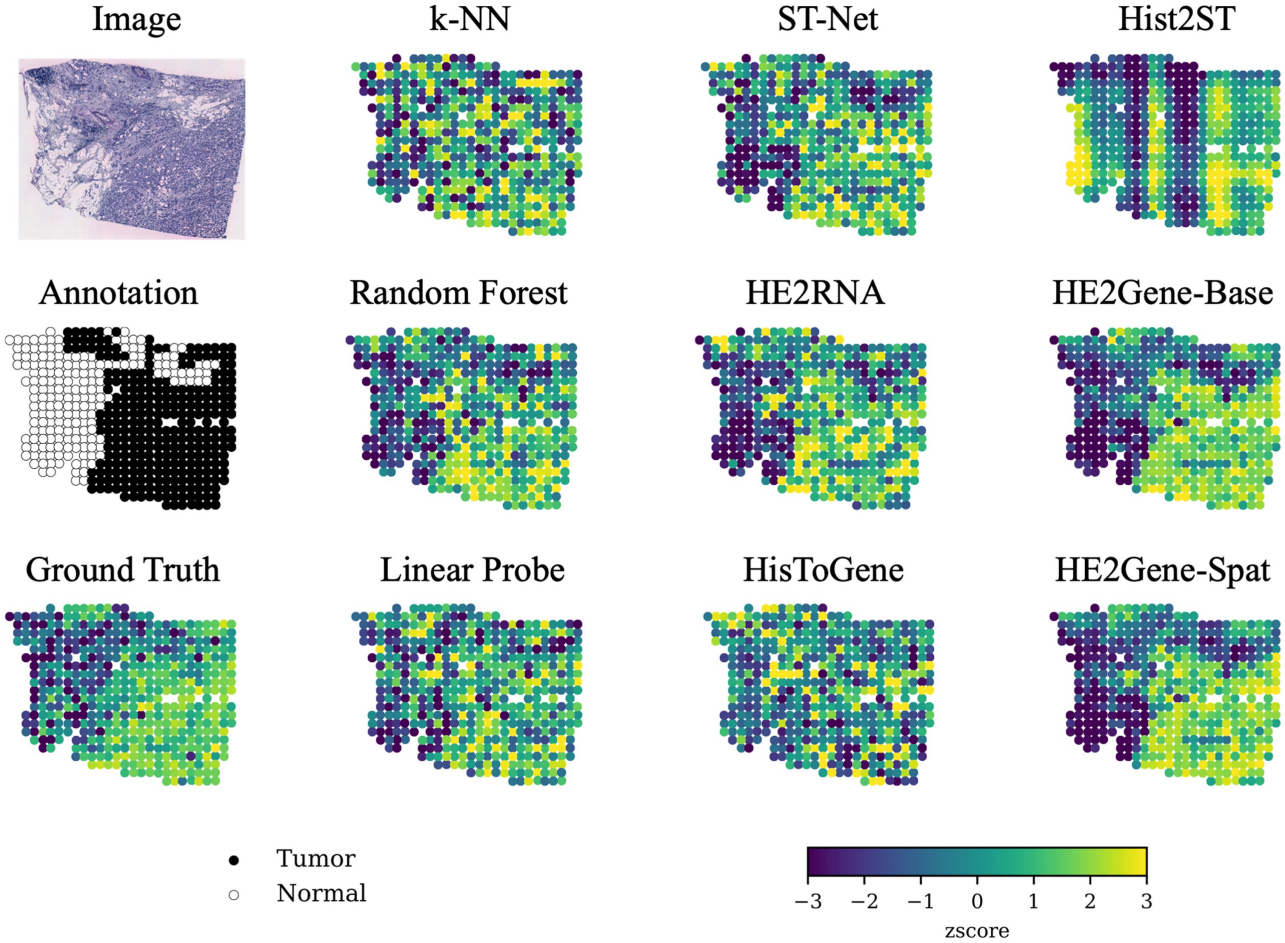
The visualization of the expression levels (in z-score) of gene H3-3B in a luminal B breast cancer patient.

We listed the average results for the top 250 target genes and the aPCCs of the top 5 genes, which were predicted with the best aPCCs on average across HE2Gene and all benchmarks (Table 2). All five genes have previously been identified as cancer biomarkers (He et al. 2020, Zheng and Fang 2022). We observed that HE2Gene outperforms all the methods in terms of aMSE, aPCC, and the number of genes with a positive correlation (NGPC). Specifically, when compared to the similar convolutional neural network (CNN)-based method ST-Net, our HE2Gene demonstrates its improvement by leveraging auxiliary tasks. HE2RNA and Linear Probe exhibit similar performance, which supports the conclusion that fine-tuning ImageNet pre-trained weights leads to significant improvements (as evidenced in Sensitivity analysis in Supplementary material). It is worth noting that our CNN-based HE2Gene shows significant advancements compared to the state-of-the-art transformer-based methods HisToGene and Hist2ST. This suggests the potential of CNN-based models to excel in fine-grained image regression tasks, surpassing the latest transformer-based approaches. In addition, we also visualized the expression levels of one of the top five genes (H3-3B) in a test patient with luminal B breast cancer (Fig. 3). We can observe that our HE2Gene is the only one that accurately predicts the gene expression in tumor spots compared with eight state-of-the-art benchmarks. This further demonstrates the complementary performance of HE2Gene and its potential in preserving annotated spatial domains.

**Table 2.** Comparison with the state-of-the-art methods for spatial gene inference.

Method	Target genes (250)			IGLL5	DDX5	ACTG1	GNAS	H3-3B
	aMSE ↓	aPCC ↑	NGPC ↑	aPCC ↑				
k-NN	1.5624	0.0812	225	0.1714	0.3287	0.2678	0.3126	0.2667
Random forest	1.4838	0.1032	226	0.2223	0.3344	0.3392	0.3462	0.3265
Linear probe	1.3536	0.1248	228	0.2302	0.3987	0.3770	0.4161	0.4099
ST-Net	1.3449	0.1315	218	0.2815	0.4145	0.3418	0.3795	0.4177
HE2RNA	1.3549	0.1313	234	0.1156	0.4015	0.3379	0.3859	0.3955
HisToGene	1.3671	0.0124	154	−0.0413	0.2046	0.1018	−0.0445	0.1070
Hist2ST	1.3534	0.0055	137	0.0076	0.0899	0.0130	−0.0421	0.0100
HE2Gene-Base	1.3336	0.1774	231	**0.4783**	**0.4483**	0.4524	**0.4969**	**0.4990**
HE2Gene-Spat	**1.3273**	**0.1818**	**237**	0.4396	0.4444	**0.4539**	0.4758	0.4880

Bold values represent the best performance.

### 3.4 HE2Gene generalizes well on external 10× genomics data without re-training

As an independent test and comparison, we applied HE2Gene to an external HER2+ dataset measured by the 10× Visium platform, which included 36 tissue sections from 8 patients (Andersson et al. 2020). Due to the limited number of spots with available manual annotations, we employed it solely as a test dataset to assess the generalization capabilities of HE2Gene. The HER2+ dataset encompassed measurements for 154 out of the 250 genes that HE2Gene was trained to predict. The data from 10× Genomics were independently generated at a different facility using a distinct staining protocol, thus providing a robust test for the reliability of HE2Gene. We divided the eight patients randomly into six for training, one for validation, and one for testing, and conducted three ablation experiments: “No Train,” “Fine-tune,” and “Train-from-scratch.” “No Train” implies that HE2Gene was directly applied to the test patient without training. “Fine-tune” involved continuing the training of HE2Gene on the HER2+ dataset, while “Train-from-scratch” entailed training the models with random initialization. For the “Fine-tune” and “Train-from-scratch” settings, we utilized the remaining 17 690 genes and seven types of pathological annotations to construct the non-target gene inference task and tumor detection task, respectively. The comparison results are available in Supplementary Fig. S8.

We can observe that the “Train-from-scratch” setting achieved the worst results compared to the three other settings. The reason may be that the size of the HER2+ dataset is too small, which prevents it from effectively addressing the batch effect/out-of-distribution problem present between training and test patients. On the other hand, it is surprising that both variants of our HE2Gene with the “No Train” setting can predict most of the genes with a positive correlation, compared with the “Train-from-scratch” model. Additionally, it is expected that the “Fine-tune” setting achieved the best performance in terms of the number of genes when compared to other settings. Furthermore, we visualized the expression of the biomarker gene ACTG1 in the test patient, as shown in Supplementary Fig. S9. These observations indicate that HE2Gene generalizes effectively to the external dataset without the need for re-training. This underscores HE2Gene’s distinctive capability and its potential for broad application across various scenarios, including those involving different staining protocols.

## 4 Discussion

In this study, we proposed HE2Gene, the first deep learning-based method that can simultaneously predict gene expressions along with spot-level pathological annotations. HE2Gene capitalizes on the relatively inexpensive and readily available whole slide imaging technology, which is a routine procedure in clinics, to accurately estimate gene expression levels. Through the integration of a multi-task learning framework, HE2Gene can predict the expressions of tens of thousands of genes alongside spot-level pathological annotations in a high-throughput manner, further eliminating the need for costly manual pathological annotations.

We evaluated HE2Gene using two breast cancer datasets, demonstrating superior performance in both gene expression prediction and tumor detection tasks compared to existing methods such as HE2RNA, ST-Net, HisToGene, and His2ST. By considering both correlations between auxiliary tasks and spatial dependencies using multi-task learning, we integrate information from non-target genes, pathological annotations, and spatial dependencies among neighboring patches for gene expression inference. The non-target gene inference task considers co-expression patterns and gene–gene interactions, while the introduced spatial constraints incorporate patch-based prior knowledge to improve predictions. Visualization analyses indicate that the gene expression levels annotated by our model preserve meaningful information. Additionally, the auxiliary task involving pathological annotations allows HE2Gene to be used not only for gene inference but also for tumor detection. We also conducted studies to assess the impact of various modeling decisions on HE2Gene. We discovered that the selection of an appropriate spot size, training strategy, and model architecture can have a significant impact on the model’s accuracy (see Sensitivity analysis in Supplementary material). Additionally, we compared the execution time of our method with that of state-of-the-art predictors (see Supplementary Fig. S10 and running efficiency in Supplementary material). While HE2Gene may not be the fastest, it achieves an ideal balance between prediction performance and running time.

Despite the advantages of HE2Gene, there are several areas where our model can be improved. For instance, the performance of the spatial constraint does not consistently yield significant improvements. We believe the reason is that manual pathological annotations are not always strictly accurate. Given that there are hundreds of spots within each slide and typically tens of slides for a ST experiment, this results in tens to hundreds of thousands of spots requiring manual annotation. Annotating at the spot level is extremely time-consuming; thus, pathologists usually annotate at the region level. While this approach saves time, it can lead to inaccuracies in annotations, negatively impacting the spatial loss. Moreover, the performance of deep learning models often depends on having a relatively large training set, yet there are few public paired image-omics datasets available in ST. To mitigate this limitation, we have employed data augmentation techniques. However, as ST advances, more training data is expected to become available in the near future, which could further enhance the performance of HE2Gene.

In summary, we demonstrate that HE2Gene provides a deep learning model for generating ST data and pathological annotations from H&E-stained images, enabling the elucidation of molecular signatures across different tissues and platforms.

## Supplementary Material

btae343_Supplementary_Data

## Data Availability

We analyzed two publicly available spatial transcriptomic datasets. The images and gene expression data are available at https://data.mendeley.com/datasets/29ntw7sh4r/5 for the HBCIS dataset and https://github.com/almaan/her2st for the HER2+ dataset.
