# Supplementary material for: HE2Gene: image-to-RNA translation via multi-task learning for spatial transcriptomics data
Source: Bioinformatics. 2024 Jun 5;40(6):btae343. doi: 10.1093/bioinformatics/btae343 (PMC11164830; doi:10.1093/bioinformatics/btae343)
Supplement: btae343_Supplementary_Data [file btae343_supplementary_data.pdf]

Supplementary Material for HE2Gene

Xingjian Chen[1,2], Jiecong Lin[3,4], Yuchen Wang[2], Weitong Zhang[2], Weidun Xie[2], Zetian Zheng[2], and Ka-Chun Wong[2,5,*]

[1] Cutaneous Biology Research Center, Massachusetts General Hospital, Harvard Medical School, Boston, MA
[2] Department of Computer Science, City University of Hong Kong, Kowloon Tong, Hong Kong SAR
[3] Molecular Pathology Unit, Center for Cancer Research, Massachusetts General Hospital, Department of Pathology, Harvard Medical School, Boston, MA
[4] Department of Computer Science, The University of Hong Kong, Pok Fu Lam, Hong Kong SAR
[5] Shenzhen Research Institute, City University of Hong Kong, Shenzhen, China
kc.w@cityu.edu.hk

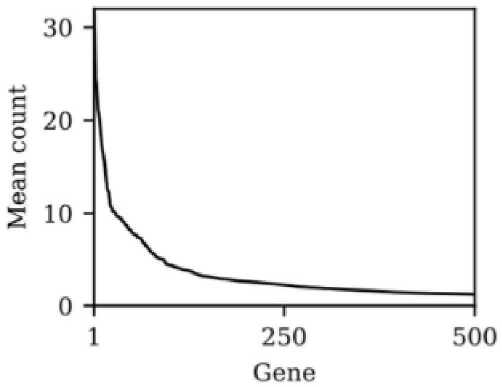

Fig. 1. The mRNA count per spot for the top 500 most expressed genes in the HBCIS dataset.

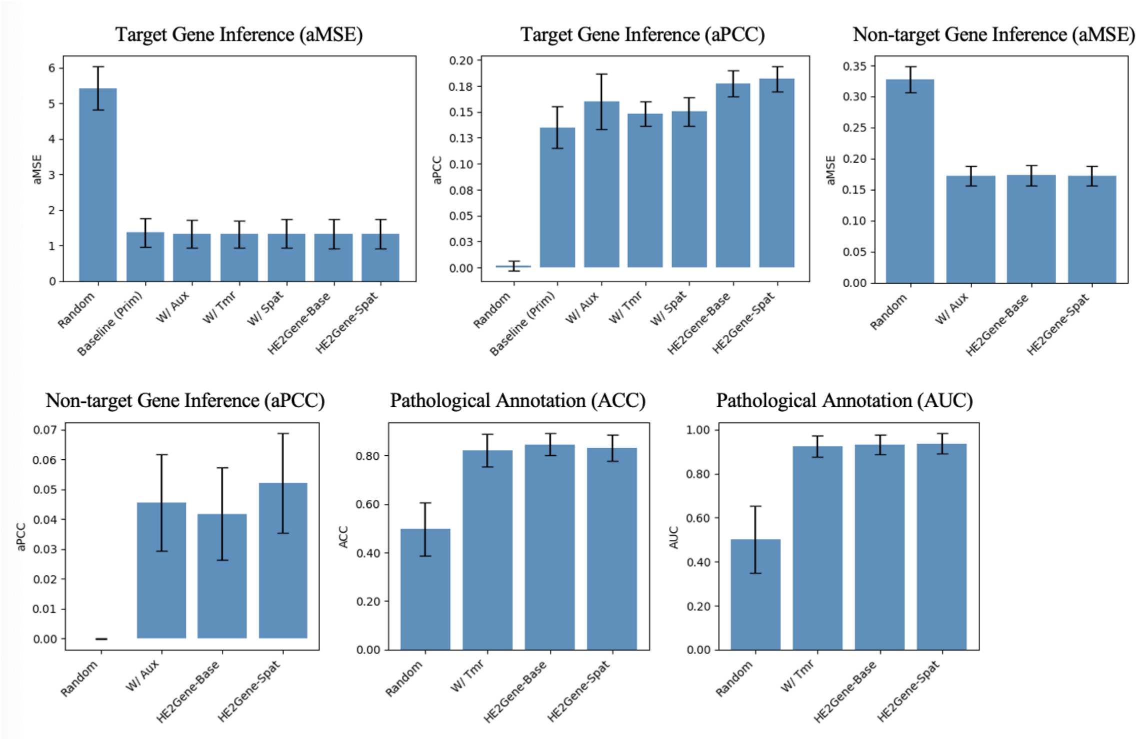

Fig. 2. Evaluation of spatial gene expression inference and pathological annotation for the HBCIS dataset. From left to right, each subfigure demonstrates the detailed results of each column in the main text Table 1. We also calculated the cosine similarity for comparison in Supplementary Table 2.

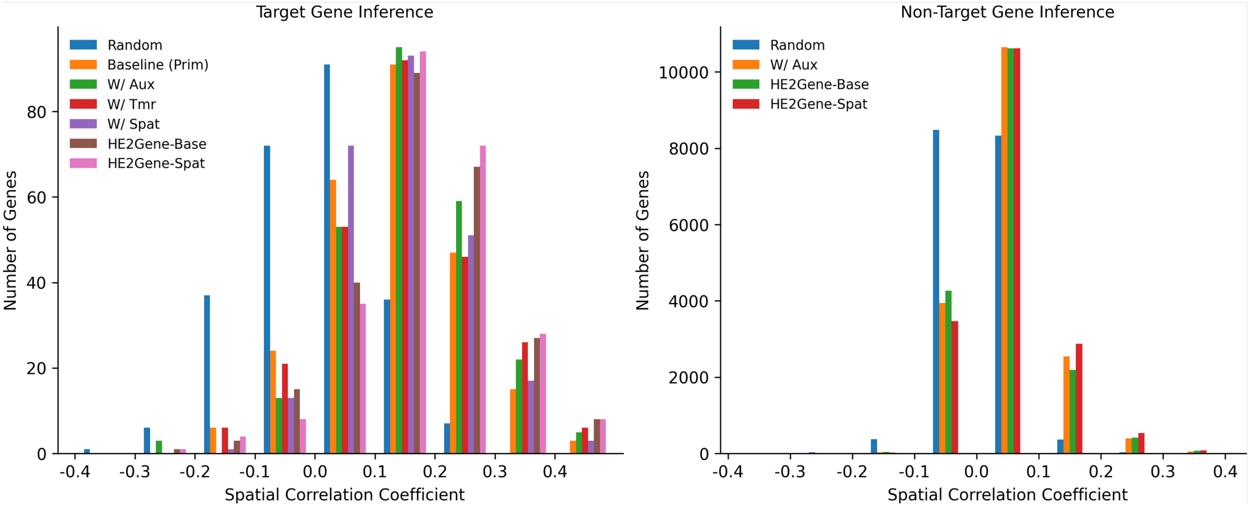

Fig. 3. Comparison of the number of genes within each interval of the spatial correlation coefficient for both target gene inference and non-target gene inference tasks. The correlation coefficient is calculated between the ground-truth gene expression and HE2Gene-predicted gene expression. A higher frequency of genes within the positive range of the correlation coefficient signifies enhanced model performance.

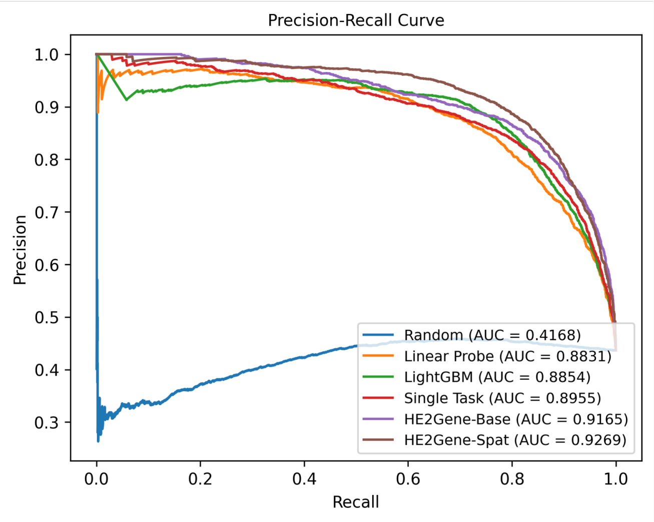

Fig. 4. The mean AUPRCs on the tumor detection task are reported. 'Random' means to predict without training. 'Linear Probe' and 'LightGBM' refer to extracting the image features from the pre-trained ResNet-50 model as the input for a linear classifier and a LightGBM for training. 'Single Task' means to separately train the model for the pathological annotation task.

Table 1. Performance comparison of the top five biomarker genes. .

Method	FASN		ACTG1		PTMA		GNAS		HSP90AB1	
	aMSE ↓	aPCC ↑	aMSE ↓	aPCC ↑	aMSE ↓	aPCC ↑	aMSE ↓	aPCC ↑	aMSE ↓	aPCC ↑
Random	4.5386	-0.1753	8.4912	-0.0148	5.3964	-0.3112	4.935	-0.0959	2.2267	0.2686
Baseline (Prim)	1.6266	0.2466	1.247	0.419	1.1616	0.2799	1.5	0.385	1.1025	0.3705
W/ Aux	1.7003	0.2959	1.1222	0.4477	1.0783	0.3783	**1.3578**	0.4698	1.0885	0.3907
W/ Tmr	**1.4995**	0.3352	1.3338	0.4343	1.1485	0.3507	1.5526	0.4268	1.0822	0.3756
W/ Spat	1.5338	0.3056	1.215	0.4026	1.1648	0.2761	1.5602	0.3686	1.0742	0.3838
HE2Gene-Base	1.5093	0.3456	1.3115	0.4524	1.1154	**0.3927**	1.4871	**0.4969**	1.0683	0.4029
HE2Gene-Spat	1.5164	**0.3582**	**1.1648**	**0.4539**	**1.0592**	0.3901	1.3833	0.4758	**1.0522**	**0.409**

Note: These top five genes exhibit the largest expression differences between tumor and normal tissue spots. The genes were selected by calculating the average gene expression of 250 target genes in both tumor and normal tissue spots across all patients and identifying the top five with the greatest differences in average expression. The selected genes—FASN, ACTG1, PTMA, GNAS, and HSP90AB1—have all been previously identified as known cancer biomarkers (Jiang et al., 2021; Kastora et al., 2022; Tripathi et al., 2011; Zhang et al., 2022; Lin et al., 2020). The positive predictive performance of HE2Gene for these genes indicates a higher correlation between biomarker genes and tissue morphology.

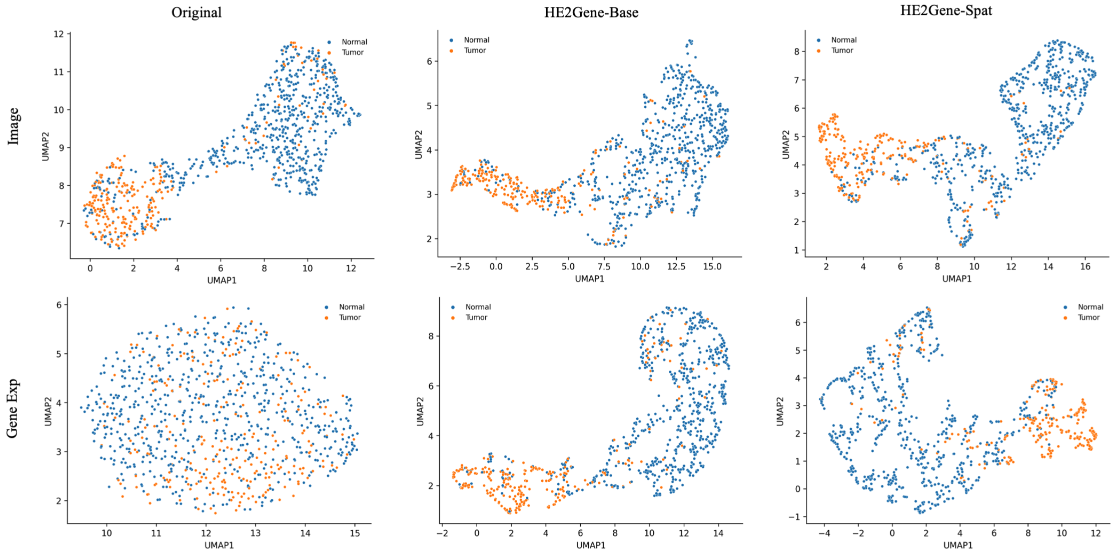

Fig. 5. Uniform Manifold Approximation and Projection (UMAP) visualization of image and gene expression embeddings for a test patient with HER2-positive breast cancer. The first column shows the original image embeddings extracted by a pretrained ResNet-50 model and gene expression embeddings based on the normalized counts. The second column shows the refined image embeddings and predicted gene expression embeddings from the HE2Gene-Base model. The third column shows the refined image embeddings and predicted gene expression embeddings from the HE2Gene-Spat model. We found that, compared to the original image embeddings extracted by the ResNet-50 model, both variants of HE2Gene have learned more meaningful and discriminatory representations for pathological annotations. Notably, whereas the original gene expression embeddings could not distinguish between normal and cancer spots, the predictions of our HE2Gene exhibited a clear decision boundary. We infer that the reason could be the multi-task framework of HE2Gene, which captures the correlation between spot phenotype and gene expression. This integration likely helps to correct the noise and batch effects in the original gene expression data, as evidenced in Supplementary Material Figure 6, thereby enhancing its efficacy for tumor detection. We also supplemented the results of Principal Component Analysis (PCA) embeddings and indicated the average silhouette width (AWI) of the normal/tumor clusters in Supplementary Figure 11.

Table 2. Comparison of cosine similarity and average Pearson correlation coefficient (aPCC) for the HBCIS dataset.

Method	Target Gene Inference (250)		Non-target Gene Inference (19699)	
	Cosine Similarity ↑	aPCC ↑	Cosine Similarity ↑	aPCC ↑
Random	0.0016	0.0013	0.0015	-0.0001
Baseline (Prim)	0.8284	0.1349	-	-
W/ Aux	0.8437	0.1602	0.2012	0.0455
W/ Tmr	0.8425	0.1482	-	-
W/ Spat	0.8415	0.1503	-	-
HE2Gene-Base	0.8541	0.1774	0.2033	0.0417
HE2Gene-Spat	0.8546	**0.1818**	**0.2041**	**0.0521**

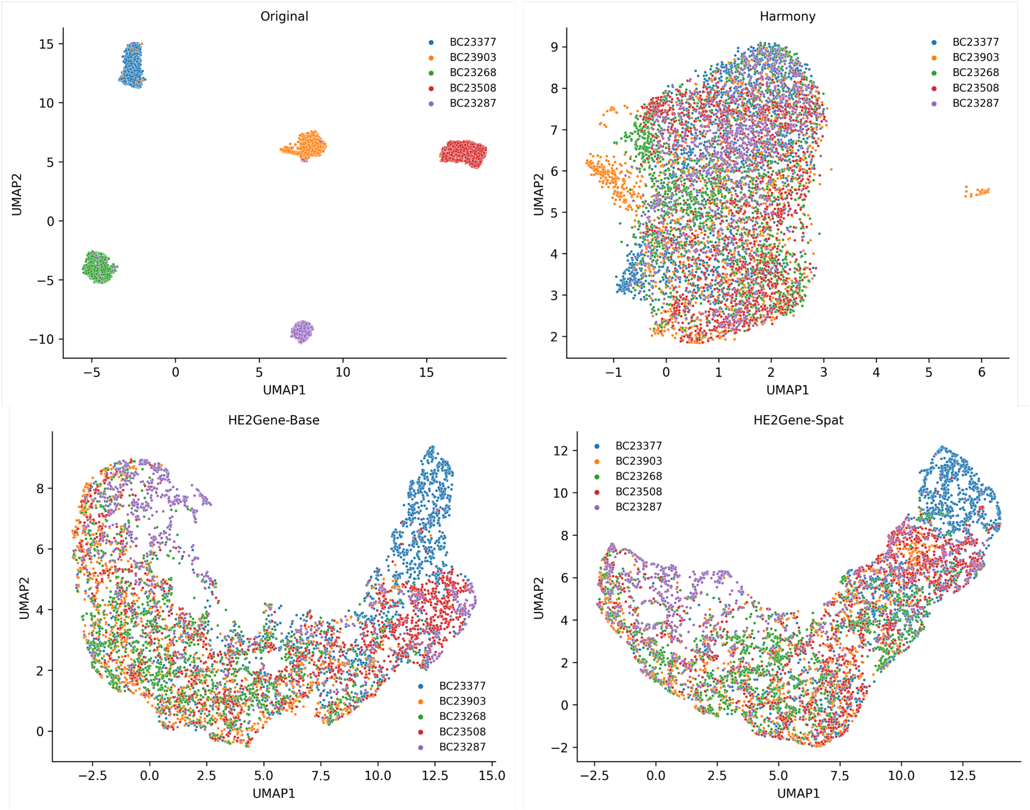

Fig. 6. UMAP visualization of the original and HE2Gene predicted gene expression embeddings across 5 test patients. Harmony is an algorithm for performing integration and removes the batch effects of scRNA-seq datasets. (Korsunsky et al., 2019).

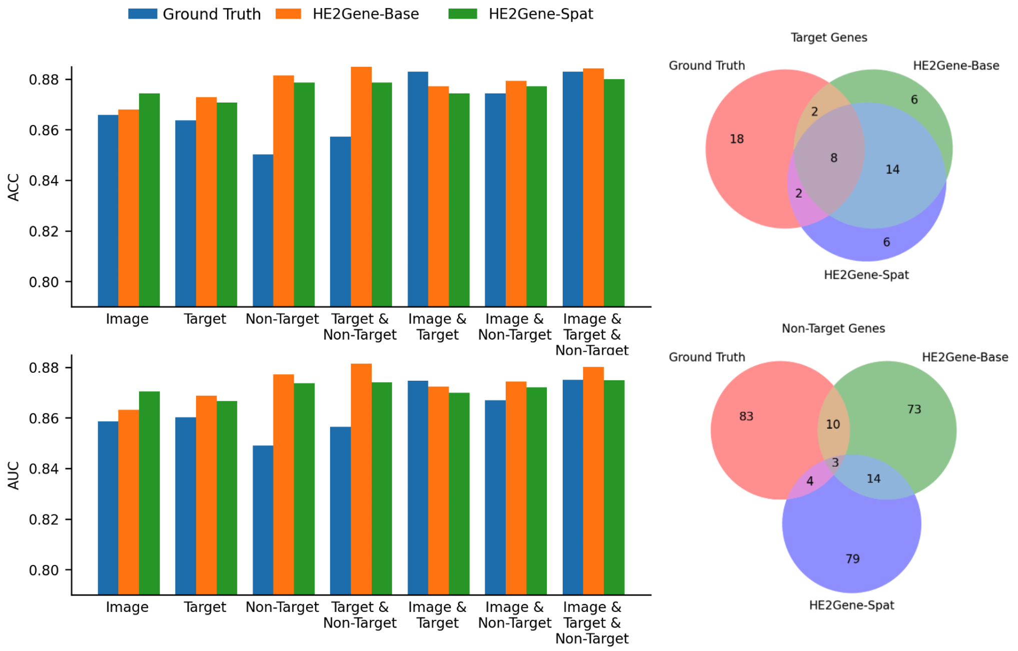

Fig. 7. [Left] Prediction results of utilizing different data modalities for the tumor detection task. We trained a random forest classifier to predict pathological annotations based on different combinations of data modalities. From left to right, each column represents the prediction results based on different modalities (or their combinations) which are image features, target gene expression features, non-target gene expression features, the concatenation of target gene expression and non-target gene expression features, the concatenation of image and target gene expression features, the concatenation of image and non-target gene expression features, and the concatenation of image and all gene expression features. The blue color (ground truth) represents the image features obtained by the original ResNet-50 and the gene expression features based on the original normalized counts. The yellow and green colors represent the predicted gene expression features and refined image features from HE2Gene-Base and HEGene-Spat. [Right] We visualize the Venn diagrams of the overlaps of the top 30 informative gene expression features extracted by the random forest trained on target gene expression features and the top 100 informative gene expression features extracted by the random forest trained on non-target gene expression features.

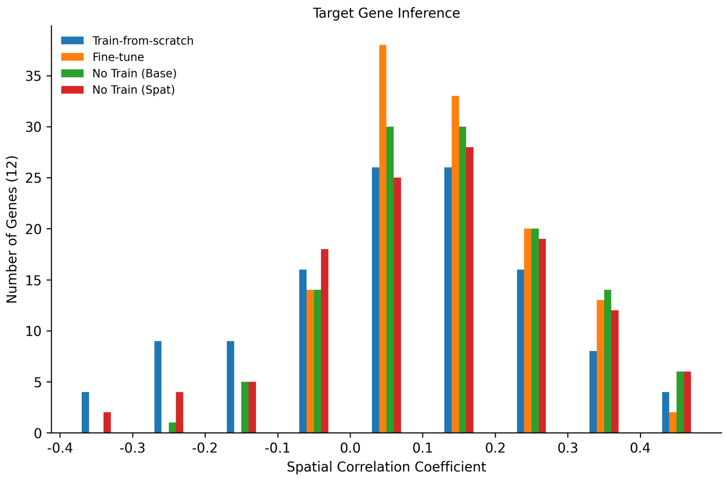

Fig. 8. Comparison of the number of genes within each interval of the correlation coefficient for target gene inference task on the HER2+ dataset. We conducted the experiments based on three different settings which were 'No Train', 'Fine-tune', and 'Train-from-scratch'. While 'No Train' means that HE2Gene-Base and HE2Gene-Spat were directly applied to predict the HER2+ test patient without training, 'Fine-tune' refers to continuing to train the HBCIS pre-trained HE2Gene on the HER2+ dataset. 'Train-from-scratch' indicates training HE2Gene from scratch with random initialization. For 'Fine-tune', and 'Train-from-scratch' settings we utilized the remaining 17,690 genes and 7 types of pathological annotations to construct the non-target gene inference task and pathological annotation task, respectively.

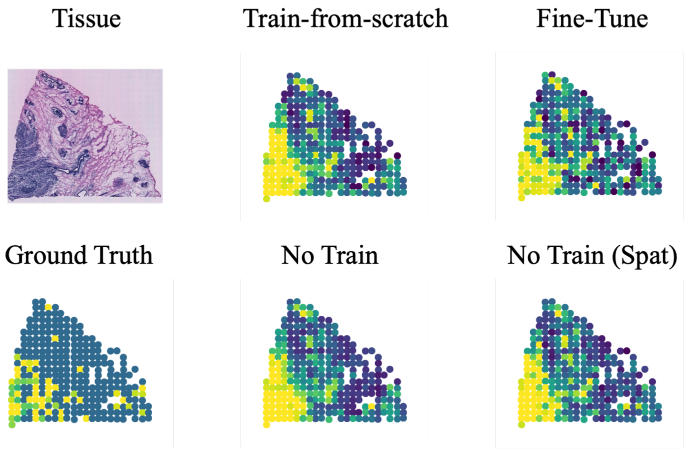

Fig. 9. The visualization of the expression of the biomarker gene ACTG1 in the HER2+ dataset. 'No Train' means that HE2Gene was directly applied to predict the HER2+ test patient without any modification 'Fine-tune' refers to continuing to train the HBCIS pre-trained HE2Gene on the HER2+ dataset. 'Train-from-scratch' indicates training the model from scratch with random initialization.

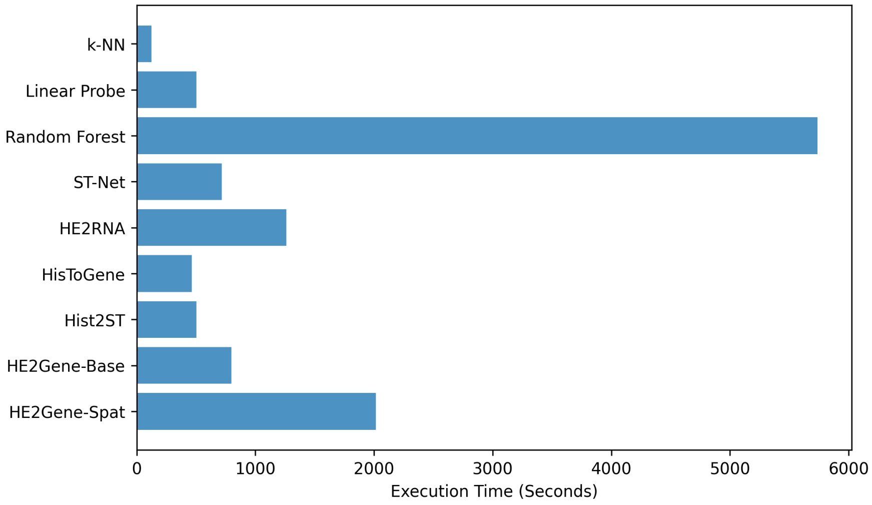

Fig. 10. Comparison of the execution time.

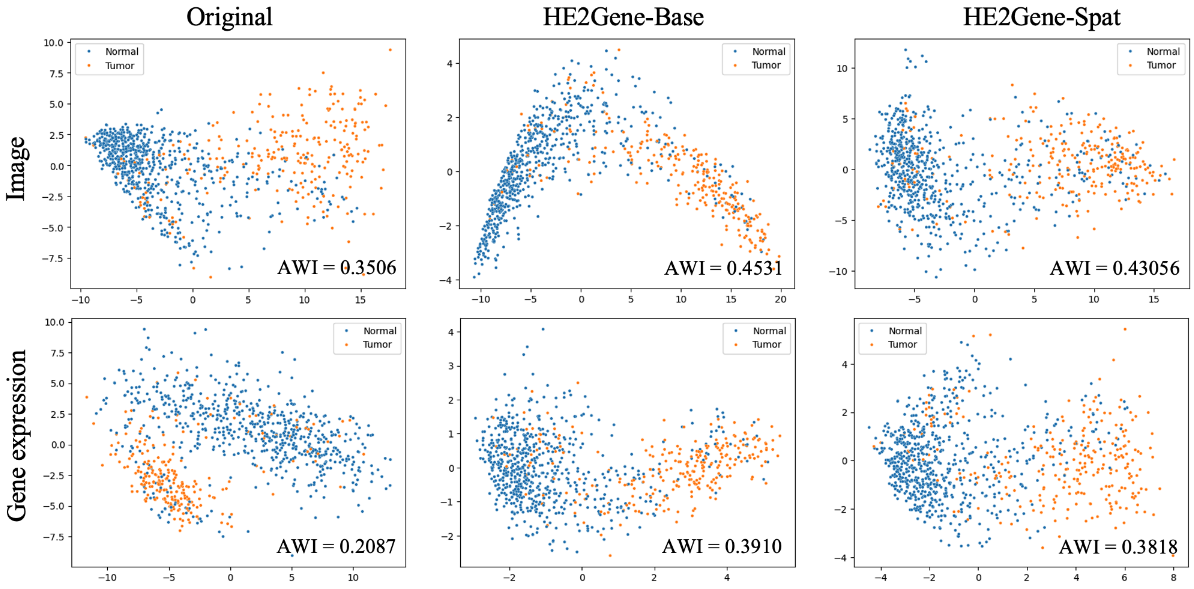

Fig. 11. Visualization of image and gene expression PCA embeddings for a test patient with HER2-positive breast cancer. AWI represents the average silhouette width of the normal/tumor clusters.

Experimental settings

The results reported in this study were conducted on Ubuntu 20.04 LTS with Intel(R) Xeon(R) CPU @ 2.20GHz, NVIDIA A100 GPU, and 256 GB memory. All experiments were implemented using the PyTorch deep learning library. To avoid bias and ensure that our predictions can generalize to different scenarios, we performed holdout validation based on the patient subtypes to evaluate the performance of gene inference. Specifically, we split the patients with a ratio of 8:2, resulting in 18 patients for training and 5 patients for testing. Instead of randomly choosing 5 test patients, we made the selection to ensure that there is at least one patient from each subtype for testing. The spots in all the sections of each patient were treated as an independent test group for evaluation, and the final prediction results are reported by calculating the average metric across all the test patients. In this study, we trained the models using the Adam optimizer with a learning rate of 1e-5 and a batch size of 256 for 100 epochs. Followed by the previous studies (He et al., 2020; Zeng et al., 2022), during training time, we augmented the dataset by randomly rotating the image by 0, 90, 180, or 270 degrees and taking the mirror image 50% of the time. During the test time, we averaged the results from each of the 8 symmetries to reduce experimental noise from spatial transcriptomics protocols. To select the best loss weights and the number of epochs, we ran an internal five-fold cross-validation on the training patients. The accuracy of gene expression inference is assessed using two evaluation metrics: average Mean Squared Error (aMSE) and average Pearson Correlation Coefficient (aPCC). These metrics are defined as following Equations 1 and 2:

$$aMSE = \frac{1}{nm}\sum_{i=1}^{n}\sum_{j=1}^{m}\left(y_{i(j)} - \hat{y}_{i(j)}\right)^2 \tag{1}$$

$$aPCC = \frac{1}{m}\sum_{j=1}^{m}\frac{\sum_{i=1}^{n}(y_{i(j)} - \bar{y}_j)(\hat{y}_{i(j)} - \bar{\hat{y}}_j)}{\sqrt{\sum_{i=1}^{n}(y_{i(j)} - \bar{y}_j)^2}\sqrt{\sum_{i=1}^{n}(\hat{y}_{i(j)} - \bar{\hat{y}}_j)^2}} \tag{2}$$

where n is the number of spots and m is the number of genes. $y_{i(j)}$ and $\hat{y}_{i(j)}$ represent the ground truth and predicted gene expression, and $\bar{y}_j$ and $\bar{\hat{y}}_j$ denote the mean of the ground truth and predicted gene expressions for gene j across all the spots. The performance of tumor detection is assessed using the Accuracy (ACC), the area under the ROC Curve (AUC), and the area under the Precision-Recall (PR) curve (AUPRC).

For deep learning-based benchmarks such as HE2RNA (Schmauch et al., 2020), ST-Net (He et al., 2020), HisToGene (Pang et al., 2021), and Hist2ST (Zeng et al., 2022), the input is the same 224 × 224-pixel RGB images as in HE2Gene, and they were all implemented using PyTorch (Paszke et al., 2019). HE2RNA utilized the ResNet-50 architecture as a CNN backbone, followed by two hidden dense layers with 1024 and 256 neurons respectively, using sigmoid activation, followed by a final prediction layer with 250 output neurons and linear activation. We trained HE2RNA with the Adam optimizer, with a learning rate of 3×10^{-4}, a minibatch size of 16, and for 50 epochs. ST-Net is initialized with pre-trained ImageNet weights from a DenseNet-121 model and trains all weights using stochastic gradient descent with a learning rate of 10^{-6} and a momentum of 0.9, for up to 50 epochs with a batch size of 32. To determine the optimal number of epochs, ST-Net performed four-fold cross-validation on the training patients. During training, data augmentation included random rotations of the images by 0, 90, 180, or 270 degrees and mirroring the images 50% of the time. For testing, we averaged the eight symmetrical outcomes from the rotations and reflections. HisToGene was implemented with the following hyperparameters: a learning rate of 10^{-5}, 100 training epochs, a dropout ratio of 0.1, eight Multi-Head Attention layers, and 16 attention heads. For Hist2ST, we set the input channels to 3, output channels to 32, kernel size to 7, and stride to 7 in the patch embedding layer of the ConvMixer module. The kernel sizes for the depthwise and pointwise convolutional blocks were set to five and one, respectively, with both input and output channels at 32. The Transformer module consisted of eight multi-head attention layers with 16 attention heads each. We configured the GNN with four layers, both input and hidden

dimensions at 1024, and the number of nearest neighbors to four. The Adam optimizer was used with a learning rate of 0.00001, and we empirically set the number of epochs to 350 for all datasets.

For traditional machine learning methods, we utilized an ImageNet pre-trained ResNet-50 model to extract 2048-dimensional image features. By using the scikit-learn (Pedregosa et al., 2011), we applied k-nearest neighbors (kNN) with 5-fold cross-validation to select the best 'n_neighbors' values from 2, 5, and 10. For logistic regression, we selected the best 'C' with 5-fold cross-validation, which is the inverse of regularization strength, from values 0.1, 0.2, 0.5, and 1.0. For random forest, cross-validation was used to determine the best 'n_estimators' from 100, 200, and 500. The default parameters for other parameters in scikit-learn were employed for training (Pedregosa et al., 2011). To ensure a fair comparison, all benchmark methods have been rigorously evaluated using the identical evaluation scheme applied to HE2Gene.

Sensitivity analysis

We further studied the effects of several modeling decisions for HE2Gene. We found that selecting an appropriate spot size, training strategy, as well as model architecture can significantly affect the accuracy of the model (See Tables 3, 4, and 5). Specifically, we found that a slightly bigger spot size will result in better and more stable performance. Additionally, fine-tuning ImageNet pre-trained weights led to substantial improvements rather than training from scratch or linear probes. Furthermore, in comparison to Transformer-based models, a CNN-based model can perform better in image-based gene inference tasks.

Table 3. Performance comparison of HE2Gene-Base with different window sizes (μm).

Window Size	aMSE ↓	aPCC ↑	NGPC ↑
50	1.3583	0.1430	208
100	1.3336	0.1774	231
150	**1.3328**	**0.1793**	**233**
200	1.3399	0.1667	225
250	1.3409	0.1658	223
300	1.3343	0.1649	221

Table 4. Performance comparison of HE2Gene-Base with different training strategies.

Strategy	aMSE ↓	aPCC ↑	NGPC ↑
Linear Probe	1.3970	0.1125	178
Fine-tuning	**1.3336**	**0.1774**	**231**
Train-from-scratch	1.3654	0.1145	183

Running efficiency

We compared the execution time of our methods with those of state-of-the-art predictors (Figure 10). Details about the development environment and implementation can be found in the Experimental Settings section. The efficiency of HE2Gene-Spat is less than optimal, as integrating the spatial-aware constraint requires the central point and its eight neighbors as input, which significantly increases GPU memory usage. Consequently, the reported time for HE2Gene-Spat is based on a batch size of 32 due to limited GPU memory, while the batch

Table 5. Performance comparison of of HE2Gene-Base with different model architectures.

Method	Architecture	aMSE ↓	aPCC ↑	NGPC ↑
CNN	VGG-16	1.3390	0.1640	223
	Inception-V3	1.3389	0.1584	218
	ResNet-50	**1.3336**	**0.1774**	**231**
	DenseNet-121	1.3650	0.1372	215
	EfficientNet-B0	1.3581	0.1306	212
	ConvNeXt-T	1.3652	0.7252	138
Transformer	ViT-B16	1.3519	0.1440	221
	Swin-T	1.3687	0.1232	215

size for other benchmarks is 256. It's unsurprising that k-NN is the fastest method, as it has no parameters to train. As for the other deep learning-based methods, our HE2Gene-Base outperforms all predictors in terms of efficiency, except for HisToGene and Hist2ST. However, these two have the worst performance across all benchmarks, as shown in Table 2. In summary, although our proposed HE2Gene is not the fastest, it may be the only method capable of achieving an ideal balance between prediction performance and running time. Additionally, it is more scalable since it can be trained on GPUs.

Bibliography

He, B., Bergenstråhle, L., Stenbeck, L., Abid, A., Andersson, A., Borg, Å., Maaskola, J., Lundeberg, J., and Zou, J. (2020). Integrating spatial gene expression and breast tumour morphology via deep learning. *Nature biomedical engineering*, 4(8):827–834.

Jiang, W., Xing, X.-L., Zhang, C., Yi, L., Xu, W., Ou, J., and Zhu, N. (2021). Met and fasn as prognostic biomarkers of triple negative breast cancer: a systematic evidence landscape of clinical study. *Frontiers in Oncology*, 11:604801.

Kastora, S. L., Kounidas, G., Speirs, V., and Masannat, Y. A. (2022). Integrative, in silico and comparative analysis of breast cancer secretome highlights invasive-ductal-carcinoma-grade progression biomarkers. *Cancers*, 14(16):3854.

Korsunsky, I., Millard, N., Fan, J., Slowikowski, K., Zhang, F., Wei, K., Baglaenko, Y., Brenner, M., Loh, P.-r., and Raychaudhuri, S. (2019). Fast, sensitive and accurate integration of single-cell data with harmony. *Nature methods*, 16(12):1289–1296.

Lin, T., Qiu, Y., Peng, W., and Peng, L. (2020). Heat shock protein 90 family isoforms as prognostic biomarkers and their correlations with immune infiltration in breast cancer. *BioMed Research International*, 2020:1–15.

Pang, M., Su, K., and Li, M. (2021). Leveraging information in spatial transcriptomics to predict super-resolution gene expression from histology images in tumors. *bioRxiv*, pages 2021–11.

Paszke, A., Gross, S., Massa, F., Lerer, A., Bradbury, J., Chanan, G., Killeen, T., Lin, Z., Gimelshein, N., Antiga, L., et al. (2019). Pytorch: An imperative style, high-performance deep learning library. *Advances in neural information processing systems*, 32.

Pedregosa, F., Varoquaux, G., Gramfort, A., Michel, V., Thirion, B., Grisel, O., Blondel, M., Prettenhofer, P., Weiss, R., Dubourg, V., et al. (2011). Scikit-learn: Machine learning in python. *the Journal of machine Learning research*, 12:2825–2830.

Schmauch, B., Romagnoni, A., Pronier, E., Saillard, C., Maillé, P., Calderaro, J., Kamoun, A., Sefta, M., Toldo, S., Zaslavskiy, M., et al. (2020). A deep learning model to predict rna-seq expression of tumours from whole slide images. *Nature communications*, 11(1):3877.

Tripathi, S. C., Matta, A., Kaur, J., Grigull, J., Chauhan, S. S., Thakar, A., Shukla, N. K., Duggal, R., Choudhary, A. R., DattaGupta, S., et al. (2011). Overexpression of prothymosin alpha predicts poor disease outcome in head and neck cancer. *PLoS One*, 6(5):e19213.

Zeng, Y., Wei, Z., Yu, W., Yin, R., Yuan, Y., Li, B., Tang, Z., Lu, Y., and Yang, Y. (2022). Spatial transcriptomics prediction from histology jointly through transformer and graph neural networks. *Briefings in Bioinformatics*, 23(5):bbac297.

Zhang, L., Sun, S., Zhao, X., Liu, J., Xu, Y., Xu, L., Song, C., Li, N., Yu, J., Zhao, S., et al. (2022). Prognostic value of baseline genetic features and newly identified tp53 mutations in advanced breast cancer. *Molecular Oncology*, 16(20):3689–3702.
